# Network pharmacology, molecular docking, and molecular dynamics simulations shed light on the mechanism behind *Gynostemma pentaphyllum’*s efficacy against osteosarcoma

**DOI:** 10.1097/MD.0000000000039454

**Published:** 2024-08-30

**Authors:** Yange Zhang, Peiyun Ji, Xiangyu Xiao, Jingshuai Wang, Zedong Wan, Haiying Cao, Lingwei Kong, Yu Jin

**Affiliations:** a Department of Traumatology and Orthopaedics, Affiliated Hospital of Chengde Medical College, Chengde, China; b Department of Obstetrics and Gynecology, Affiliated Hospital of Chengde Medical College, Chengde, China.

**Keywords:** *Gynostemma pentaphyllum* (Thunb.), molecular docking, molecular dynamic, network pharmacology, osteosarcoma

## Abstract

Osteosarcoma (OS) is the most common type of malignant bone tumor, that poses a serious threat to the lives and health of children and adolescents. Traditional Chinese medicines (TCM) have gained attention for treating OS because of their potent anti-cancer effects and fewer side effects. It is commonly understood that *Gynostemma pentaphyllum* (Thunb.) Makino (GP) exhibits inhibitory effects on most tumors. However, the knowledge of the systematic mechanisms involved is limited. In this study, the Traditional Chinese Medicine Systems Pharmacology Database and Analysis Platform (TCMSP) was searched to screen the effective ingredients and corresponding target genes of GP, and disease target databases were searched to identify relevant targets for OS. Venn analysis was used to visualize overlapping genes, which were further extracted using the protein-protein interaction network. R software was used to conduct gene ontology and Kyoto encyclopedia of genes and genomes pathway enrichment analysis, molecular docking and molecular dynamics simulation further validate the binding efficacy of potential therapeutic targets to compound molecules. In total, 161 and 1981 proteins were identified as target genes of GP and OS, respectively, and 104 overlapping genes were identified. Through analysis of the core subnetwork, 12 hub genes were identified, and Kyoto Encyclopedia of Genes and Genomes pathway enrichment analyses revealed that the PI3K/Akt signaling pathway was the most significant. Molecular docking and molecular dynamics simulations show that a high affinity between quercetin and these targets, especially with the combination of TNF free energy (Δ Gbind) minimum, MM/PBSA and MM/GBSA is 42.85 kcal/mol, respectively, and 45.29 kcal/mol. The active ingredients Rhamnazin and Quercetin in Gypenoylum play a therapeutic role in OS through several key targets and pathways. This study provides ideas and references for further research on drug development.

## 1. Introduction

Osteosarcoma (OS) stands as the most common type of primary malignant bone tumor. Its incidence demonstrates a bimodal distribution, with peaks typically occurring at 18 and 60 years of age, with a slight male predilection towards OS development.^[1]^ OS is recognized for its intricate heterogeneity and the abnormal production of immature osteoid matrix,^[2–4]^ rendering it the prevalent malignant bone-related cancer among young individuals. Current treatment modalities for OS encompass neoadjuvant chemotherapy, surgical resection, and adjuvant chemotherapy.^[5,6]^ Despite concerted efforts by researchers, there has been negligible progress in improving the 5-year survival rate of OS patients over recent decades, underscoring the inadequacy of current therapeutic approaches.^[7–9]^ Moreover, these methods often fall short in effectively eradicating all OS cells due to challenges in drug delivery precision, particularly concerning metastatic and circulating OS cells, thus predisposing to tumor recurrence and progression.^[10,11]^ Hence, there is an urgent imperative to explore innovative therapeutic strategies for OS. Furthermore, acquired drug resistance commonly manifests in OS patients, concomitant with metastasis or recurrence in distant organs. The overall 5-year cumulative survival rate remains dismally low at 20%, with a persistently high recurrence rate of approximately 35%.^[12]^

Traditional Chinese medicine (TCM) is gaining traction as a complementary cancer treatment. TCM has been found to bolster immune function, optimize the tumor microenvironment, and mitigate the adverse effects of radiotherapy and chemotherapy, thereby enhancing overall survival rates.^[13]^ Furthermore, several clinical studies have demonstrated that the integration of Chinese medicine with chemotherapy not only circumvents drug resistance but also attenuates chemotherapy-related side effects.^[14]^ In clinical practice, there is an urgent need for safe and efficacious drugs that are readily accessible and devoid of drug resistance. Medicinal plants offer promising alternatives for cancer treatment.^[15]^ *Gynostemma pentaphyllum* (Thunb.) Makino (*G. pentaphyllum*, GP), commonly known as jiaogulan in Chinese, is a trailing herb belonging to the Cucurbitaceae family native to China. Since the Ming Dynasty, it has been extensively utilized as a dietary supplement and herbal infusion.^[16]^ Jiaogulan (*G. pentaphyllum*) was first documented in the Herbal for the Relief of Famines. Its earliest recorded medicinal applications date back to the Compendium of Materia Medica, which detailed its efficacy in treating conditions such as hematuria, edema, sore throat, fever, neck edema, tumors, and traumatic injuries. Subsequently, the herb garnered widespread recognition, leading to extensive studies on its therapeutic properties to support its traditional medicinal use.^[17]^ In recent years, systematic research has been undertaken to investigate the chemical composition, pharmacology, and clinical and healthcare applications of Thunb.^[18]^

**Figure 1.**
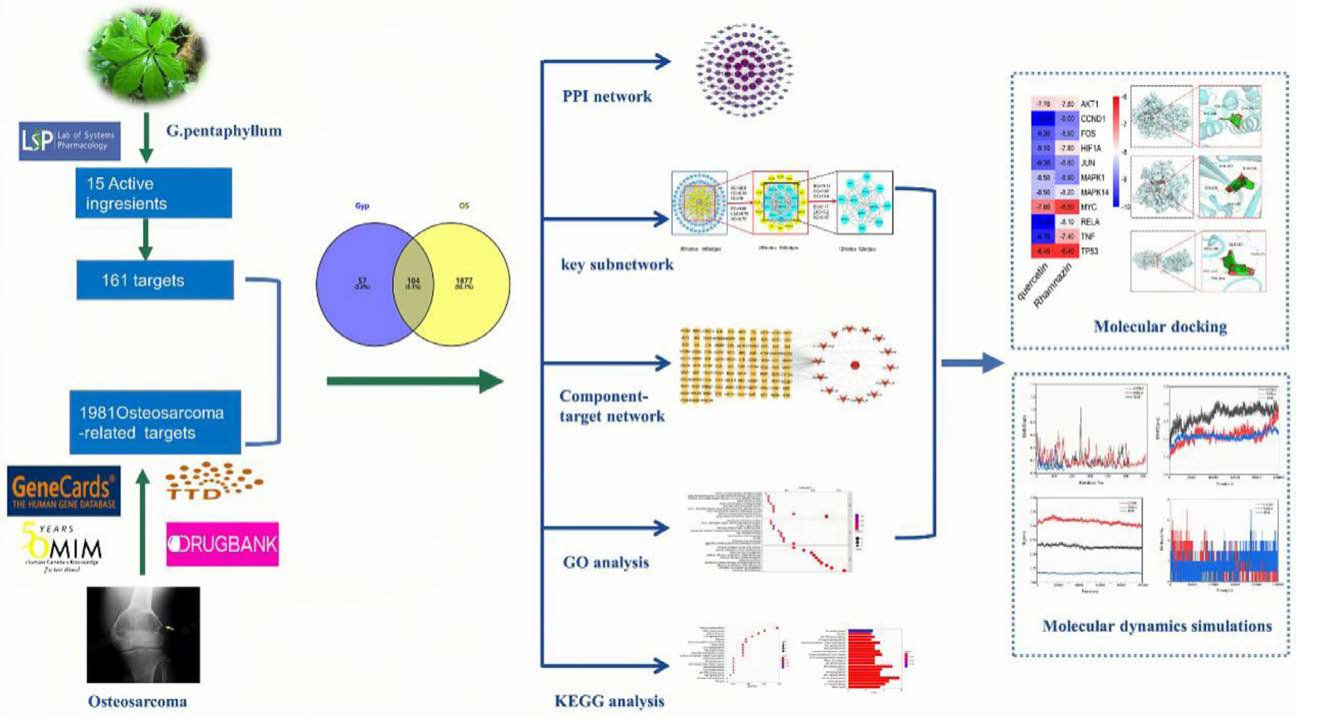
The workflow diagram of network pharmacology study on the anti-OS cell activity of *Gynostemma pentaphyllum* (GP).

Network pharmacology employs static algorithms to integrate extensive datasets from large databases, facilitating the exploration of interactions among diverse drug components, targets, and disease mechanism.^[19,20]^ It is widely acknowledged that network pharmacology explores the regulatory impact of drugs on the biomolecular interactome from a systems perspective, akin to the holistic approach of Traditional Chinese Medicine (TCM). Consequently, it is being utilized to probe potential therapeutic targets and pathways for GP in OS treatment. Molecular docking is a virtual screening technique used to precisely locate binding sites and measure the strength of interaction between drugs and their targets. This method reveals molecular interactions and predicts binding patterns and affinities. Additionally, molecular dynamics simulations are employed to closely observe the movement and interactions of molecules over time, providing intricate insights into molecular conformation and dynamics to validate the reliability of molecular docking. The workflow of this study was shown in Figure 1.

## 2. Materials and methods

### 2.1. Screening for active ingredients and prediction of targets

The active ingredients of GP were screened by searching the Traditional Chinese Medicine Systematic Pharmacology (TCMSP) platform (http://old.tcmsp-e.com/tcmsp.php), comprehensive data related to the pharmacokinetic properties of each chemical compound were provided for the screening and evaluation of the compounds. The specific screening criteria used were based on oral availability (OB) ≥ 30% and drug-likeness (DL) ≥ 0.18. The targets of the active ingredients that met these criteria were identified using TCMSP. The screened target proteins were uniformly transformed into coding gene symbols using UniProt protein database (https://www.uniprot.org).

### 2.2. The collection of potential targets for OS

We used “osteosarcoma” as keyword to query the following four databases: GeneCards Human gene database (GeneCards) (https://www.genecards.org/), Online Mendelian Inheritance in Man (OMIM) (https://www.omim.org/), DrugBank database (https://go.drugbank.com), and Therapeutic Target Database (http://db.Idrblab.net/ttd/). To acquire the final disease target information, the retrieved target proteins were standardized using UniProt database (https://www.uniprot.org/) and transformed into Gene Symbol. Targets were obtained by merging the findings and removing duplicates.

### 2.3. Identification of cross-target genes

The collected drug and disease targets were de-duplicated and Venn plots were created using the online tool Venn 2.1.0 (https://bioinfogp.cnb.csic.es/tools/venny/index.html), which is an interactive tool for comparing lists with Venn diagrams. Overlapping genes were used for the subsequent analyses.

### 2.4. Drug – active ingredient – disease target network

A tabular formula was used to determine the core ingredients of the drug that acted on the target. The key compounds and intersecting targets were imported into Cytoscape 3.9.1 software to build a compound-target network and analyze the network nodes.

### 2.5. Network of protein-protein interactions

To evaluate the interaction relationship between drug targets of GP and disease targets of OS, protein–protein interaction network (PPI) network maps were created by importing overlapping genes into the String 11.5 (https://cn.string-db.org/) online platform. the lowest interaction threshold for the input target was set to “Maximum confidence,” while the other parameters were left as default. The species was set to “Homo sapiens.” The network diagram was imported into Cytoscape 3.7.1 software for further network analysis and image processing.

### 2.6. Identification of core targets of GP in the treatment of OS

The tsv file of protein interactions on the online String platform was imported into the Cytoscape 3.9.1. Using the CytoNCA plug-in in Cytoscape software, the following six metrics were identified for network topology analysis: Degree centrality, topology intermediateness, closeness centrality, feature vector centrality, local average connectivity, and network centrality. The 6 indicators deeply analyze the attributes of all nodes in the interactive network; the higher the quantization value, the more important it is. A target was selected only if all 6 attribute values exceeded their respective median values.^[19]^ The optimal core network is selected using the method described above. This step was implemented in the R language. Based on network topology parameters, the central target and its corresponding active ingredients were determined for subsequent molecular docking.

### 2.7. GO and KEGG enrichment analysis and visualization

Gene ontology (GO) and Kyoto Encyclopedia of Genes and Genomes (KEGG) enrichment analyses and visualization of intersection genes were performed using R packets such as “ClusterProfiler,” “org.Hs.e.g..db,” and “ggplot2” in R. The gene annotation information came from “org.Hs.e.g..db,” the adjusted *P* value cutoff value is .05, and the q value cutoff value during enrichment is .05. The corresponding bubble and bar charts represent the outputs.

### 2.8. Molecular docking

In this study, Autodock tool 1.5.6 and Pymol software were utilized to modify the structures of receptors and ligands, and the 2D structures of the principal active ingredients were downloaded from the PubChem database (https://pubchem.ncbi.nlm.nih.gov) and saved in the SDF file format. Chem3D software was used to convert the image to the SDF format, optimize its 3D structure, and save it in mol2 format. The 3D structures of the key active ingredients were converted into PDBQT file format using the AutoDock tool. The crystal structures of the targets were downloaded from the PDB database and processed using PyMOL software. This included the removal of water molecules and hydrogenation, and saved them in the pdbqt format. Processed macromolecular protein receptors and small-molecule ligands were introduced into the AutoDock Vina software for molecular docking. Finally, PyMOL software was used to evaluate the binding activity of the key active ingredients and targets, and to observe and analyze the docking results of the compound with the protein.

### 2.9. Molecular dynamics simulation

GROMACS (2021.03) software was used to perform 100ns molecular dynamics simulations of core components and key action targets to minimize the error between the conformation obtained by docking proteins with small molecules and the actual complex. The amber99sb-ildn force field was selected to build the protein topology file. A truncated octahedral TIP3P solvent box was added at a distance of 10 nm, and Na/Cl was added to the system to balance the system charge. The energy was minimized by using 2500 step steep descent method and conjugate gradient method. Under the conditions of a temperature of 298.15 K and a pressure of 101.325 kPa, the system gradually heats up under NVT (constant volume constant temperature) and NPT (constant pressure constant temperature). Finally, under the periodic boundary condition, a 100 ns molecular dynamic ensemble simulation was carried out, the integration step was 2 seconds, and the tracks were saved every 10 ps. After the simulation, root-mean-square deviation and root-mean-square fluctuation (RMSD/RMSF), as well as cyclotron radius (Rg) and the change in the number of hydrogen bonds (H-bonds), were assessed using GROMACS software. The stability of protein binding to ligand was analyzed. Meanwhile, the binding free energy was calculated by using MM/GBSA and MM/PBSA methods by extracting stable molecular conformations of equilibrium trajectories.

### 2.10. Statistical analysis

All statistical analyses in this study were performed using one-way ANOVA and graphs were plotted using R software (version 4.3.1). A threshold of *P* < .05 was used as the threshold for a statistically significant difference.

## 3. Results

### 3.1. Screening of active compounds and potential targets

The active ingredients of GP were identified by screening the Pharmacology Platform for Traditional Chinese Medicine Systems platform (http://old.tcmsp-e.com/tcmsp.php). The specific screening criteria were oral availability (OB) ≥ 30% and drug-likeness (DL) ≥ 0.18, and 24 active ingredients were collected. Among these, 15 accurately matched the target protein (Table 1); thus, 161 active ingredient targets of the drug were obtained.

**Table 1:** Information on 15 carefully selected key bioactive compounds of *Gynostemma pentaphyllum* (GP).

Mol ID	Molecule Name	OB (%)	DL
MOL000098	quercetin	46.43	0.28
MOL000338	3′-methyleriodictyol	51.61	0.27
MOL000351	Rhamnazin	47.14	0.34
MOL000359	sitosterol	36.91	0.75
MOL000953	CLR	37.87	0.68
MOL004350	Ruvoside_qt	36.12	0.76
MOL004355	Spinasterol	42.98	0.76
MOL005438	campesterol	37.58	0.71
MOL005440	Isofucosterol	43.78	0.76
MOL009855	(24S)-Ethylcholesta-5,22,25-trans-3beta-ol	46.91	0.76
MOL009867	4α,14α-dimethyl-5α-ergosta-7,9(11),24(28)-trien-3β-ol	46.29	0.76
MOL009877	cucurbita-5,24-dienol	44.02	0.74
MOL009878	Cyclobuxine	84.48	0.7
MOL009971	Gypenoside XXVII_qt	30.21	0.74
MOL009973	Gypenoside XXVIII_qt	32.08	0.74

DL = drug-likeness, OB = oral bioavailability.

### 3.2. Acquisition of disease-related target

Four databases were queried using the keyword “osteosarcoma” to search for genes related to OS. In the GeneCards database, a higher score indicates a closer relationship between the target and disease. Based on experience, if there are numerous targets, those with a score greater than the median are typically considered as potential targets for OS. Finally, 1981 target genes of OS were obtained after removing duplicates.

### 3.3. PPI network analysis and screening of hub genes

**Figure 2.**
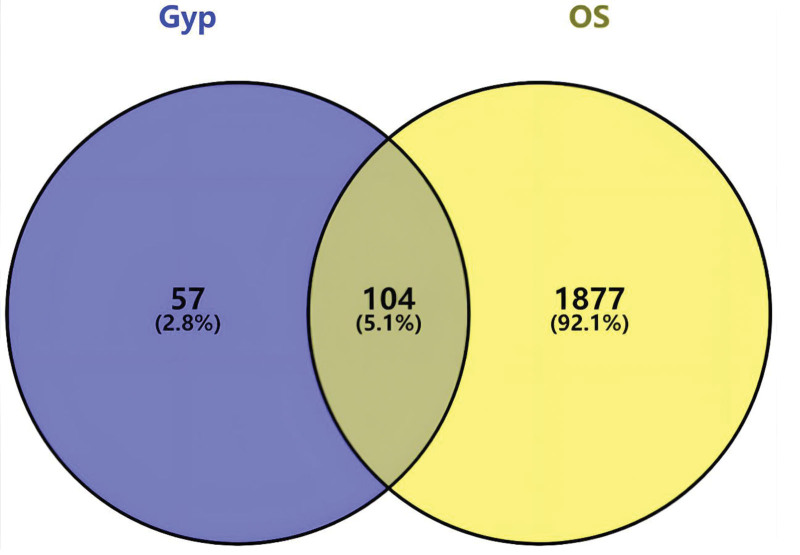
Common targets were identified by taking an intersection of drug-target genes and OS-related genes. The yellow circle stands for the targets of GP, The Blue circle stands for the targets of OS, the intersection of the two circles stands for the target of GP and OS. GP = *Gynostemma pentaphyllum* (Thunb.) Makino, OS = osteosarcoma.

**Figure 3.**
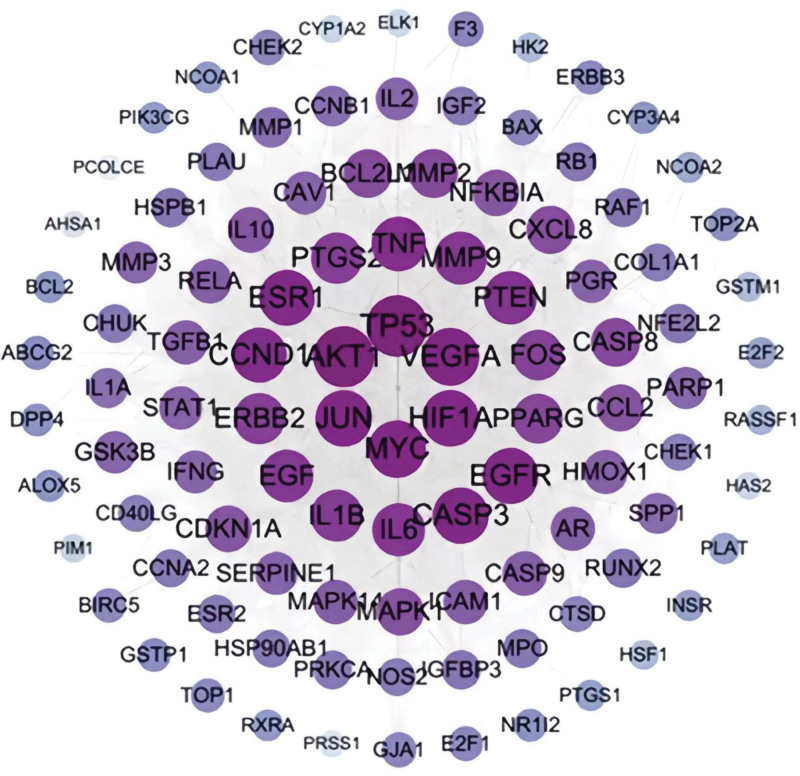
Protein–protein interaction (PPI) network, bigger node sizes and darker purple colors indicate a higher degree of association.

**Figure 4.**
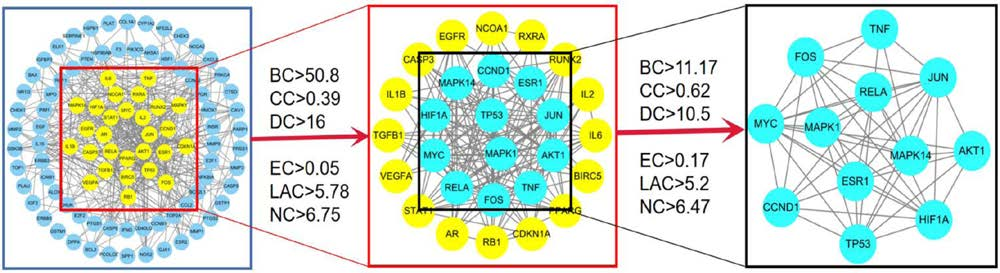
Identification of a critical PPI subnetwork for drug-target interactions. (A) We applied the above methods to screen the core subnetwork. The interactive PPI network of common targets includes 99 nodes and 446 edges. (B) PPI network of significant target proteins extracted from A; this network comprises 28 nodes and 152 edges. (C) PPI network of significant proteins extracted from B; this network is made up of 12 nodes and 52 edges. BC = betweenness centrality, CC = closeness centrality, DC = degree centrality, EC = eigenvector centrality, NC = network centrality, LAC = local average connectivity, PPI = protein-protein interaction.

A Venn diagram was constructed using 161 component targets and 1981 OS-related genes, and 2 intersections were chosen to achieve a common goal, as shown in Figure 2. A PPI network of overlapping genes was constructed, and the results are shown in Figure 3. The network includes 189 nodes, 859 edges, and an average node degree of 9.09. The interaction information for each protein was automatically scored; the higher the score, the more reliable the protein interaction. The results of the topology analysis showed that the target proteins with high degree values included TP53 (tumor protein p53), AKT1, JUN, MYC, and HIF1A, Abased on the PPI network of intersection targets. We screened the network using the key attribute values of the nodes and obtained the following core subnetwork after 2 rounds of screening, as shown in Figure 4.

### 3.4. Network diagram of drug-compound-target

**Figure 5.**
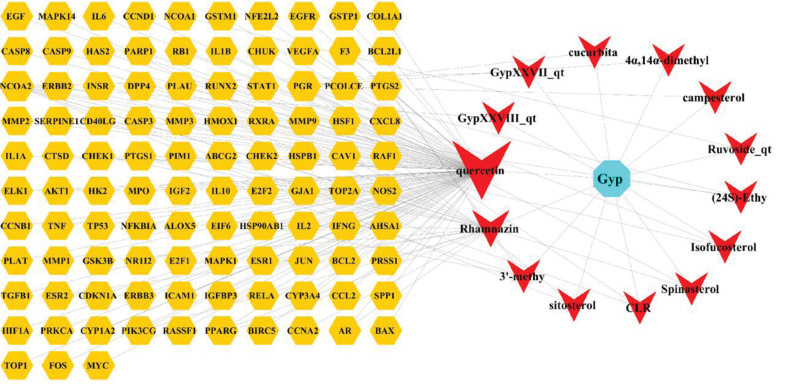
Network diagram of drug-ingredients-targets, the blue pentagon node red V-shaped triangle nodes stand for active ingredients of GP, and orange pentagon nodes represent the shared targets of GP and OS. the size of nodes is proportional to degree centrality by topology analysis. 3′-methyl = 3′-methyleriodictyol, (24S)-ethy = (24S)-Ethylcholesta-5,22,25-trans-3beta-ol,4α,14α-dimethyl = 4α,14α-dimethyl-5α-ergosta-7,9(11),24(28)-trien-3β-ol, Cucurbita = Cucurbita-5,24-dienol, GP = *Gynostemma pentaphyllum* (Thunb.) Makino, GypXXVII_qt = Gypenoside XXVII_qt, GypXXVIII_qt = Gypenoside XXVIII_qt, OS = osteosarcoma.

To uncover the interactions between the herb constituents and potential targets in the treatment of OS, the active ingredients that could act on the intersection targets were screened and a component target network was constructed based on 14 compound components and 104 intersecting targets, as shown in Figure 5. The size of the compound composition nodes represents their degree of action on disease target genes.

### 3.5. GO function and KEGG pathway enrichment analysis

**Figure 6.**
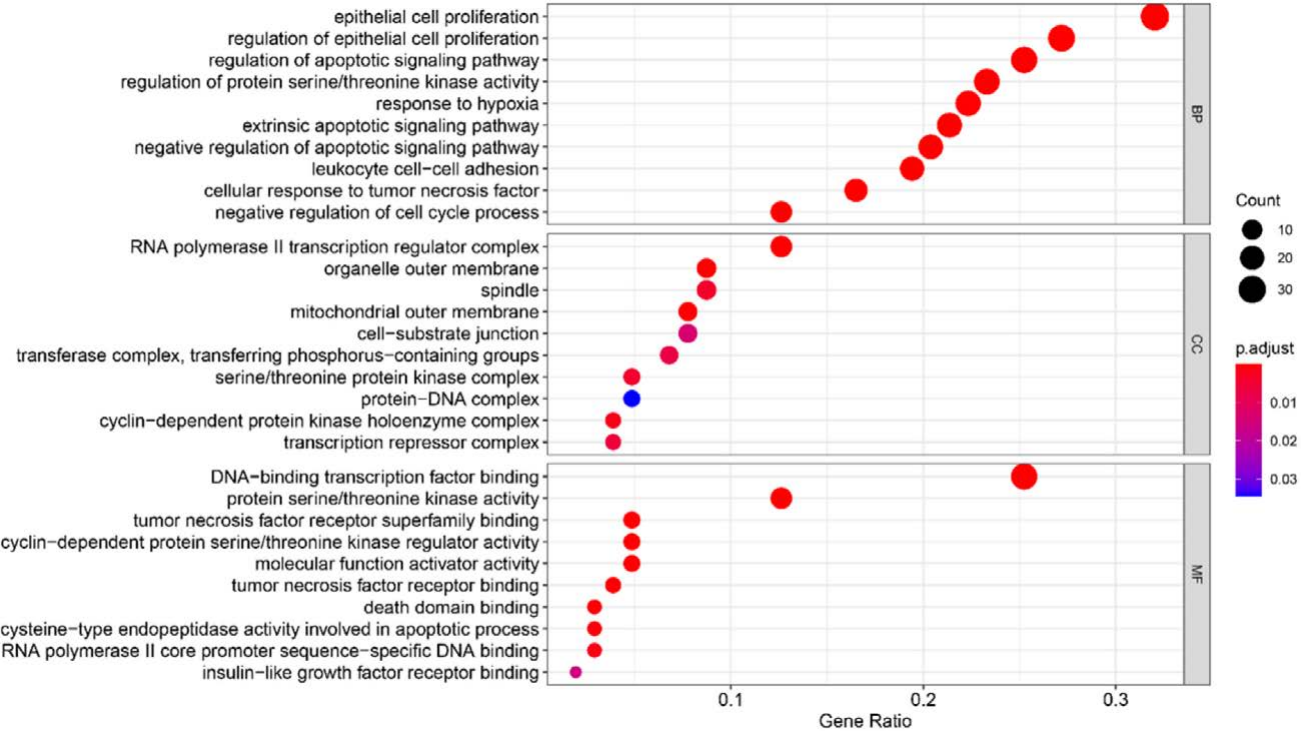
GO (Gene Ontology) biological function analysis results, which are displayed in a bubble plot.

**Figure 7.**
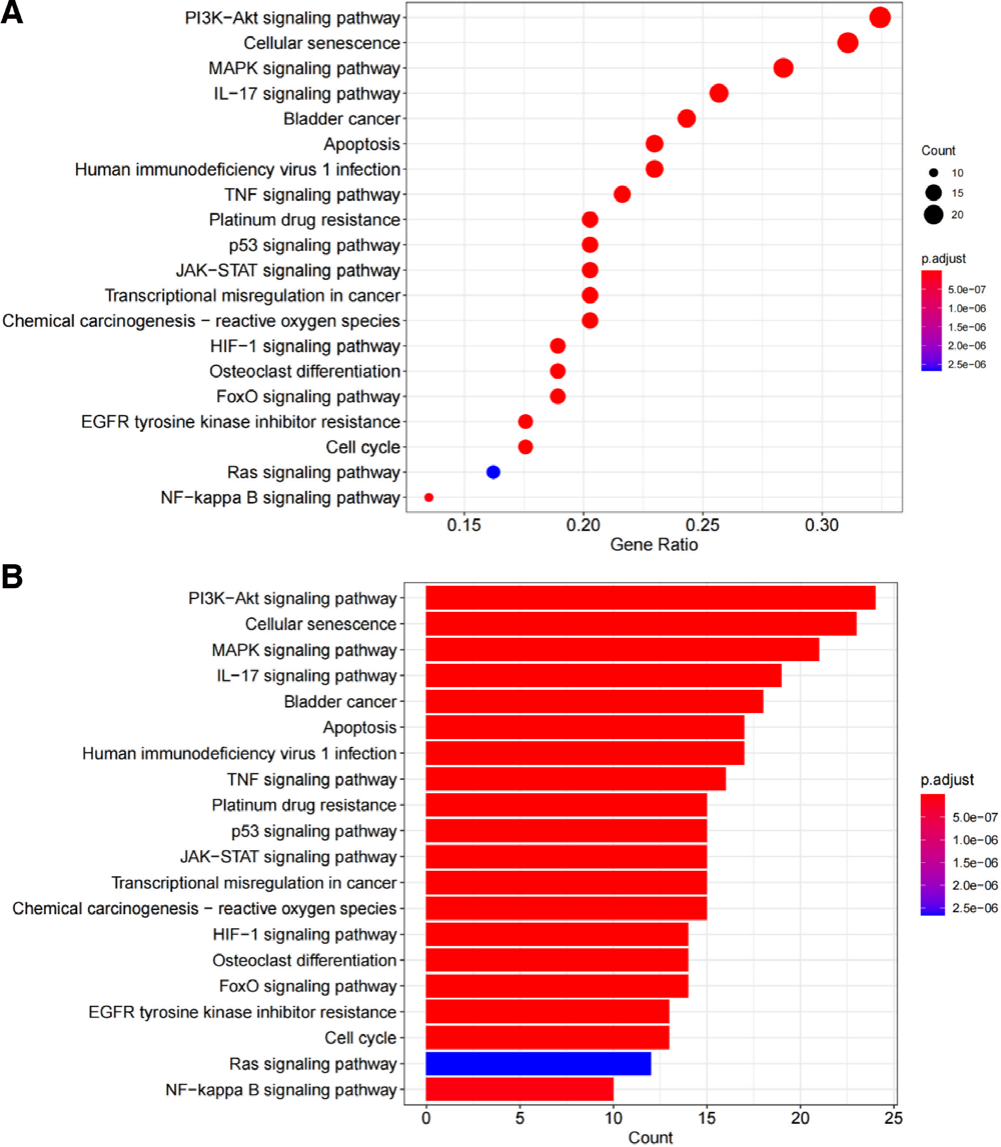
KEGG pathway enrichment analysis for the targets in GP treating OS (*P* value < .05). The color scale indicates the different thresholds of the adjusted *P* value, and the size of the point indicates the gene count of each item. (A) Bubble diagram of KEGG pathway enrichment analysis (B) The barplot of KEGG enrichment. GP = *Gynostemma pentaphyllum* (Thunb.) Makino, KEGG = Kyoto Encyclopedia of Genes and Genomes, OS = osteosarcoma.

We further investigated the potential mechanisms of action of these genes in OS. GO and KEGG enrichment analyses were conducted using R software (https://www.r-project.org/). The results of GO analysis showed that the key target proteins were predominantly located in cellular components, such as organelles and the cytoplasm. These targets are primarily involved in biological processes, such as the response to hypoxia, regulation of apoptotic signaling pathways, cell response to tumor stimulation, and regulation of epithelial cell proliferation. The molecular function of these targets is primarily related to protein binding. The top 10 entries for each GO category are shown in Figure 6. In this study, given a significance level of *P* < .05, we screened the signaling pathways most related to tumorigenesis, the results suggested that these genes were distinctly enriched in the PI3K–AKT signaling pathway, the MAPK signaling pathway, cellular senescence, apoptosis-related signaling pathway, and osteoclast differentiation. The bar chart and bubble diagram were constructed based on the selection of the top 20 terms (Fig. 7A and B).

### 3.6. Validation of molecular docking

**Figure 8.**
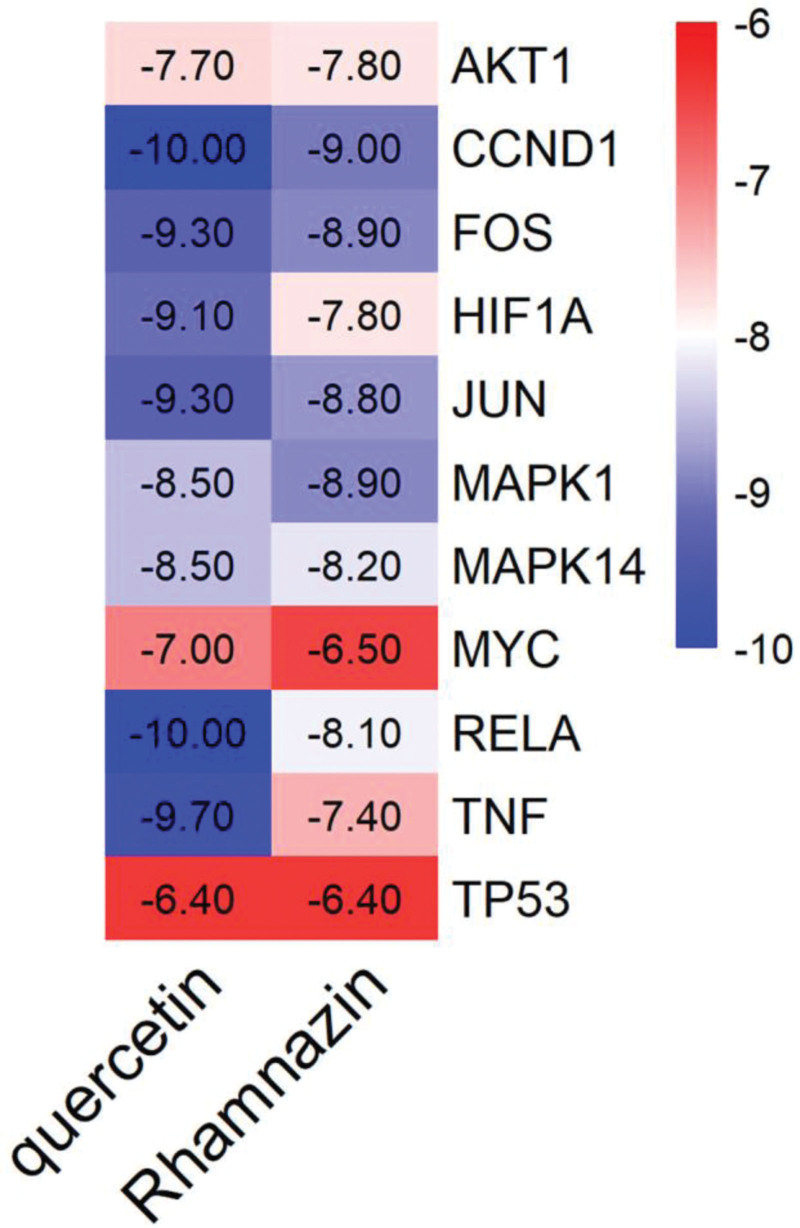
A heat map depicting the binding affinity of the active compound to the core target was generated via molecular docking. Heat maps are created using the binding energy fraction as a basis. The darker the shade of blue, the lower the binding energy score, indicating a stronger affinity between the bioactive ingredient and the core target, leading to closer binding.

**Figure 9.**
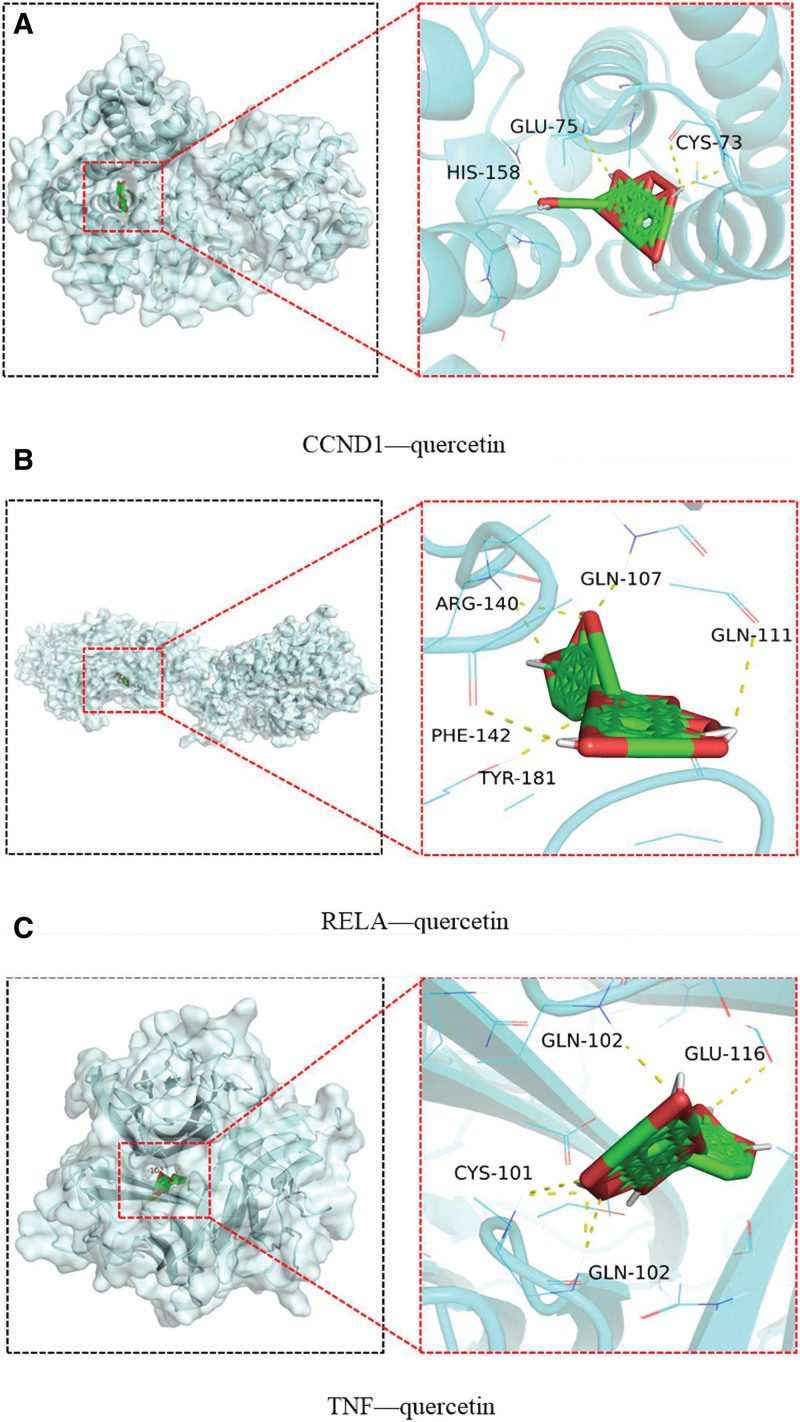
Visualization of the docking and binding of quercetin with three core targets. (A) quercetin-CCND1; (B) quercetin-RELA; (C) quercetin-TNF.

To validate the robust binding activity between the core target and its respective active ingredient, we conducted further molecular docking analysis. Twelve core targets obtained by PPI network screening were used as receptors. The specific information of protein receptor screening is shown in Table 2. Reverse matching is performed on the components corresponding to the target, and combining the ingredient-target network and the above matching results, we obtained 2 main core components as shown in Table 3. Molecular docking was performed between the obtained protein receptor and the corresponding active ingredient, and the binding strength was evaluated according to the docking score, and the binding efficiency is shown in Figure 8 below. The scoring criteria for binding energy are as follows: binding energy <0 kcal/ mol indicates that the compound has the ability to bind to the protein, binding energy <−5.0 kcal/mol indicates that the compound is well bound to the protein, binding energy <−7.5 kcal/mol indicates that the compound is well bound to the protein. As shown in Figure 9, The first three ligand receptor complexes with the lowest binding energy were selected for study. The binding affinity between quercetin and CCND1 is −10 kcal/mol, with both compounds forming hydrogen bonds at the amino acid residues HIS-158, GLU-75, and CYS-73 (Fig. 9A). Quercetin binds to RELA with a binding energy of −10 kcal/mol, establishing hydrogen bonds at the amino acid residues GLN-111, GLN-107, TYR-181, Phe-142, and Arg-140 (Fig. 9B). The binding energy between quercetin and TNF is −9.7 kcal/mol, with hydrogen bonds formed at the amino acid residues GLN-111, GLN-107, TYR-181, Phe-142, GLN-102, GLU-116, GLN-102, and CYS-101 (Fig. 9C).

**Table 2:** The related information about the 12 core targets.

Rank	Name	DC	BC	CC	EC	LAC	NC
1	JUN	21	80.3468853	0.818181818	0.315841645	8.380952381	17.258948
2	FOS	17	46.39818507	0.72972973	0.261927307	7.058823529	11.62395521
3	MAPK1	17	46.21498835	0.72972973	0.266188949	6.823529412	10.7217033
4	MAPK14	16	40.50871263	0.710526316	0.253248185	6.75	10.11959707
5	TP53	16	30.67457212	0.710526316	0.259704769	7.5	11.16478799
6	RELA	15	23.86865079	0.692307692	0.249322534	7.733333333	11.07423965
7	AKT1	14	24.83382669	0.675	0.216486797	5.857142857	8.213888889
8	MYC	14	20.43135387	0.675	0.244060755	7.571428571	9.671289821
9	HIF1A	14	24.29950901	0.675	0.230224758	6.857142857	9.368908869
10	ESR1	13	11.6613072	0.658536585	0.233859479	7.384615385	8.707792208
11	CCND1	12	13.79316913	0.642857143	0.202914804	6.333333333	8.038419913
12	TNF	11	14.22452848	0.627906977	0.171864942	5.454545455	7.180952381

BC = betweenness centrality, CC = closeness centrality, DC = degree centrality, EC = eigenvector centrality, NC = network centrality, LAC = local average connectivity.

**Table 3:** Two active components in line with the main objective.

Mol ID	Molecule name	Chemical formula	InChIKey	Canonical SMILES
MOL000098	Quercetin	C15H10O7	REFJWTPEDVJJIY-UHFFFAOYSA-N	C1 = CC(=C(C = C1C2 = C(C(=O)C3 = C(C = C(C = C3O2)O)O)O)O)O
MOL000351	Rhamnazin	C17H14O7	MYMGKIQXYXSRIJ-UHFFFAOYSA-N	COC1 = CC(=C2C(=C1)OC(=C(C2 = O)O)C3 = CC(=C(C = C3)O)OC)O

### 3.7. The study of molecular dynamics simulation

**Figure 10.**
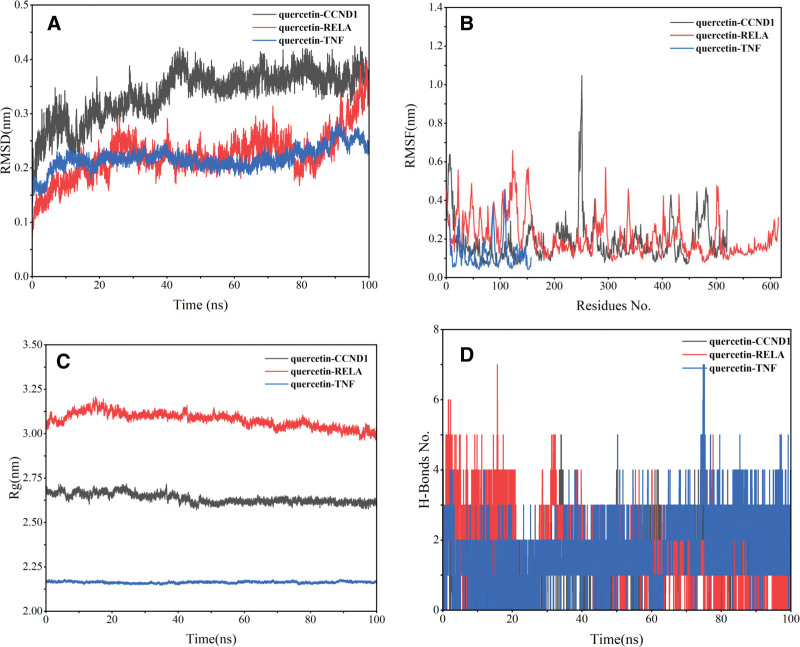
The molecular dynamics simulation trajectories over 100 ns simulation time. (A) root mean square deviation of the C-alpha atoms; (B) root mean square fluctuation; (C) radius of gyration of the complex; and (D) hydrogen bonds of the complex (the black curve represents CCND1-quercetin, the red curve represents RELA-quercetin, and the blue curve represents TNF-quercetin).

We initially conducted RMSD and RMSF analyses to assess system stability and amino acid flexibility. Figure 10A depicts RMSD values over time for the three systems. By 40 ns, all systems reached stability: the CCND1-quercetin complex remained at 0.35 nm, the RELA-quercetin complex stayed at 0.2 nm before rising to 0.35 nm, and the TNF-quercetin complex remained at 0.2 nm. These findings highlight quercetin’s stability upon binding to these proteins, particularly TNF. Subsequent RMSF analysis (Fig. 10B) revealed relatively low values (mostly between 0.1 and 0.6 nm) for the three protein complexes. However, the CCND1 complex exhibited greater fluctuation compared to RELA and TNF, particularly near residue base 250, indicating a highly flexible region. This suggests CCND1 and its complex domains are inherently more flexible. Comparison of amino acid residue fluctuations showed minor changes in TNF protein dynamics in the presence of quercetin, indicating stronger binding. The radius of rotation (Rg) confirmed tighter binding, consistent with other indicators (Fig. 10C), indicating quercetin enhances protein tightness. Fluctuations in the number of hydrogen bonds during the simulation process indicate conformational changes and structural rearrangements. As illustrated in Figure 10D, the TNF-quercetin complex exhibits a relatively higher number of hydrogen bonds compared to the other 2 complex systems, suggesting a more stable and compact structure in their binding. Calculations of total binding free energy (Tables 4 and 5) via MM/PBSA and MM/GBSA methods yielded −45.29 and −42.85 kcal/mol, respectively, underscoring quercetin’s importance in targeted therapy for OS.

**Table 4:** The binding free energy of quercetin with three core targets was estimated using MM-PBSA (kcal/mol).

No	ΔE_VDW_ (Kcal/mol)		ΔE_ELE_ (Kcal/mol)	ΔE_gas_ (Kcal/mol)	ΔG_GB_ (Kcal/mol)	ΔG_Esurf_ (Kcal/mol)	ΔG_solv_ (Kcal/mol)	ΔG_bind_ (Kcal/mol)
CCND1		−21.64	−17.44	−39.08	24.32	−3.24	21.08	−18.00
RELA		−14.32	−34.96	−49.28	33.47	−3.12	30.35	−18.93
TNF		−51.62	−21.43	−73.05	35.44	−7.68	27.77	−45.29

**Table 5:** The binding free energy of quercetin with three core targets was estimated using MM-GBSA (kcal/mol).

No	ΔE_VDW_ (Kcal/mol)	ΔE_ELE_ (Kcal/mol)	ΔE_gas_ (Kcal/mol)	ΔG_GB_ (Kcal/mol)	ΔG_Esurf_ (Kcal/mol)	ΔG_solv_ (Kcal/mol)	ΔG_bind_ (Kcal/mol)
CCND1	−20.56	−17.17	−37.73	26.75	−2.88	23.87	−13.85
RELA	−13.36	−34.63	−48.26	31.52	−2.79	28.73	−19.58
TNF	−52.53	−20.05	−75.58	36.12	−6.39	29.73	−42.85

## 4. Discussion

OS is a primary malignant bone tumor with a poor prognosis and high mortality rates. It is the most common clinical condition affecting the health of children and adolescents. Owing to early metastasis of OS, surgical intervention is often not a viable option for eliminating sarcoma cells.^[18]^ Therefore, it is critical to identify potential adjuvant therapies for improving patient survival. Over the past few years, GP has attracted considerable interest because of its diverse biological effects and minimal pharmacotoxicity.^[20]^ Initially, GP was primarily consumed as tea for its pleasurable taste and effectiveness in terms of weight loss. Over time, GP has been recognized as more than just a drink. A variety of bioactive compounds, such as saponins (also known as gypenosides, GPSs), flavonoids, polysaccharides (GPPs), and phytosterols, have been isolated and identified in GP, indicating their substantial medical value. Both in vivo and in vitro tests, of different cell lines and animal models, have demonstrated that GP possesses a variety of biologically active compounds, such as anti-cancer, anti-atherogenic, anti-dementia, and anti-Parkinson’s effects along with lipid-regulating properties, neuroprotection, hepatoprotection, and hypoglycemic activity.^[21]^ Although GP has medicinal value above, the underlying mechanism remains unclear. Considering these issues and the complex heterogeneity of OS, we investigated new therapeutic approaches that involve a range of molecular targets. The analysis of this pathogenic mechanism provides a promising avenue for future research on OS treatment.^[22]^ *Gynostemma pentaphyllum* (Thunb.) Makino belongs to the Cucurbitaceae family and is commonly referred to as “Jiaogulan” in China. It is a climbing plant that thrives in China, Korea, Japan, and other Southeast Asian regions annually. It is widely used as a folk medicine owing to its anti-cancer, anti-hyperlipidemic, anti-hyperglycemic, and immuno-modulatory effects.^[23]^ Systematic research has been conducted in recent years on the chemical composition, pharmacology, clinical applications, and health care development of this plant. To date, approximately 180 gypenosides have been isolated from *G. pentaphyllum*. GP possesses multiple therapeutic properties. For instance, in line with TCM theory, it can regulate the flow of qi, fortify qi, and nourish blood. Best recognized as an herbal remedy and has demonstrated efficient treatment of various ailments owing to its anti-cancer^[24]^ and anti-inflammatory properties.^[25]^ In vitro studies have shown that GP plays a pivotal role in tumor treatment through multiple pathways and various biological processes. However, the biological mechanisms that underlie OS remain unclear.

In terms of the results of the current network pharmacology analysis, we successfully identified and explained the potential pharmacological activity and mechanism of GP, and uncovered all core targets of GP against OS.^[26]^ comprising ESR1, TP53, MYC, JUN, RELA, FOS, HIF1A, MAPK1, CCND1, AKT1, and TNF. In particular, anti-OS activity was revealed by identifying the biological functions of these core targets, which were distinctly enriched in regulatory processes such as cellular transcription activity, epithelial cell proliferation, and regulation of the apoptotic signaling pathway. Therefore, the viability of OS cells can be influenced, to a certain extent, by regulating these key targets. Mutations in TP53 have also been identified in the pathway. TP53, also known as p53, is encoded by the TP53 gene on human chromosome 17. It acts as a tumor suppressor gene in the human body and protects genomic integrity. Tumor protein p53 (TP53) functions as a tumor suppressor, through multiple mechanisms of anti-cancer activity. TP53 plays a role in apoptosis, genomic stability, and angiogenesis inhibition. TP53 mutations are associated with tumorigenesis and carcinomatous metastasis.^[27]^ A previous study showed that TP53 mutations occurred in 47% to 90% of patients with OS and negatively impacted the 2-year overall survival. Therefore, targeting TP53 may be an effective strategy for OS treatment.^[18]^ MYC is a commonly expressed oncogene in OS and is associated with both metastasis and an unfavorable prognosis.^[28]^ The amplification of the 8q24 chromosomal locus, where the oncogene MYC is located, has been documented in multiple patients with OS. A recently created MYC knock-in mouse model for OS not only detected inherent MYC-mediated mechanisms of OS tumorigenesis but also revealed a new molecular mechanism by which MYC governs the composition and operation of the OS immune ecosystem.^[29]^ As observed in the results of the KEGG pathway enrichment analysis, many other diseases were also enriched together with OS, which may be due to the presence of the same molecular targets in different diseases. We selected signaling pathways closely related to OS for further analysis. Oncogenic signaling pathways mainly involve the PI3K-Akt signaling pathway, MAPK signaling pathway, apoptosis, osteoclast differentiation, and tumor necrosis factor TNF signaling pathway. As an important signaling pathway, the MAPK signaling pathway plays a key role in the occurrence and development of OS. The JUN protein is a key signaling node in this pathway. JUN is a transcription factor, also known as AP-1, that consists of a heterodimer of Fos and Jun proteins, both of which are encoded by proto-oncogenes. It plays a crucial role in the regulation of cell proliferation, differentiation, transformation, and apoptosis. The activation of there proteins is intricately involved in these processes.^[30]^ The active components of GP play key regulatory roles in OS by acting on these targets. Currently, the PI3K/AKT signaling pathway has received much attention in cancer research. It has been found that the PI3K/AKT signaling pathway is disrupted in most human tumors. AKT1, a well-known oncogene, is phosphorylated by PI3K to activate the PI3K/AKT signaling pathway. AKT1 encodes a protein kinase that plays a crucial role in the PI3K signaling pathway and is involved in regulating cell viability and apoptosis. Mutations in AKT1 activate kinase activity and stimulate downstream signal transduction, which results in OS. Multiple studies have shown that the inhibition of AKT1 may also inhibit osteoclastogenesis.^[31]^ The PI3K/AKT signaling pathway, activated by HIF-1α, regulates glycolysis in hypoxic environments. Inhibition of the PI3K/AKT pathway can significantly reduce the expression level of glycolysis, which may have potential therapeutic value in the future.^[32]^

In this analysis of targets enriched in osteoclast differentiation-related pathways, we noticed that were many genes, including the core genes, RELA and MAPK1, were enriched in the signaling axis of osteoclast differentiation. The RELA proto-oncogene, which is a subunit of NF-kB and also referred to as P65, is a widely distributed transcription factor that plays a crucial role in various biological processes. It has been demonstrated that RELA directly targets MAPK1 and sustains NF-kB activation. Therefore, both MAPK1 and RELA may serve as potential pharmacological targets in OS. At the same time, we found that these core genes were also enriched in multiple other important signaling pathways; therefore, it is reasonable to assume that GP exerts anti-OS effects through multiple targets and pathways.

Finally, we present the molecular docking results of quercetin with three pivotal targets, accompanied by molecular dynamics simulations. These findings suggest that the interaction of quercetin with these proteins is plausible, potentially identifying it as a promising target for OS treatment. However, it is crucial to acknowledge the limitations of this study. Firstly, the results were obtained from literature and databases, thus their reliability and accuracy are contingent upon data quality. Secondly, the lack of evidence to substantiate our conclusions underscores the need for in vitro and in vivo experiments to validate these findings. Thirdly, experimental validation is essential to further confirm quercetin’s affinity towards key targets. Until computational predictions are corroborated by experimental validation, our conclusions remain preliminary.

## 5. Conclusions

In this study, we explored the molecular mechanisms of GP in the treatment of OS using network pharmacology, molecular docking, and molecular dynamics simulations. Quercetin and Rhamnazim are the 2 most important active components of GP anti-OS. By acting on CCND1, RELA, HIF1A, JUN, TNF, and other targets, GP regulates several signaling pathways, including PI3K/Akt, MAPK, cell aging, and TNF signaling pathway. Promoting cell apoptosis, regulating cell cycle and regulating cell response to tumor necrosis factor contribute to its anti-OS effect. Molecular docking and dynamic simulation showed that the binding between CCND1-quercetin, RELa-quercetin and TNF-quercetin was more stable and tight.

## Author contributions

**Conceptualization:** Yange Zhang, Zedong Wan.

**Data curation:** Xiangyu Xiao, Jingshuai Wang, Zedong Wan, Haiying Cao.

**Formal analysis:** Haiying Cao, Lingwei Kong, Yu Jin.

**Investigation:** Jingshuai Wang.

**Methodology:** Peiyun Ji, Xiangyu Xiao, Lingwei Kong.

**Project administration:** Haiying Cao, Yu Jin.

**Resources:** Xiangyu Xiao, Jingshuai Wang, Zedong Wan.

**Software:** Peiyun Ji, Xiangyu Xiao, Zedong Wan.

**Supervision:** Haiying Cao, Lingwei Kong, Yu Jin.

**Visualization:** Yange Zhang, Peiyun Ji.

**Writing – original draft:** Yange Zhang.

**Writing – review & editing:** Lingwei Kong, Yu Jin.
